# Phenotypic and Genotypic Characteristics of Novel Mouse Cell Line (NIH/3T3)-Adapted Human Enterovirus 71 Strains (EV71:TLLm and EV71:TLLmv)

**DOI:** 10.1371/journal.pone.0092719

**Published:** 2014-03-26

**Authors:** Carla Bianca Luena Victorio, Yishi Xu, Qimei Ng, Vincent T. K. Chow, Kaw Bing Chua

**Affiliations:** 1 Temasek Lifesciences Laboratory, 1 Research Link, National University of Singapore, Singapore; 2 Host and Pathogen Interactivity Laboratory, Department of Microbiology, Yong Loo Lin School of Medicine, National University Health System, National University of Singapore, Singapore; The University of Hong Kong, Hong Kong

## Abstract

Since its identification in 1969, Enterovirus 71 (EV71) has been causing periodic outbreaks of infection in children worldwide and most prominently in the Asia-Pacific Region. Understanding the pathogenesis of Enterovirus 71 (EV71) is hampered by the virus’s inability to infect small animals and replicate in their derived *in vitro* cultured cells. This manuscript describes the phenotypic and genotypic characteristics of two selected EV71 strains (EV71:TLLm and EV71:TLLmv), which have been adapted to replicate in mouse-derived NIH/3T3 cells, in contrast to the original parental virus which is only able to replicate in primate cell lines. The EV71:TLLm strain exhibited productive infection in all primate and rodent cell lines tested, while EV71:TLLmv exhibited greater preference for mouse cell lines. EV71:TLLmv displayed higher degree of adaptation and temperature adaptability in NIH/3T3 cells than in Vero cells, suggesting much higher fitness in NIH/3T3 cells. In comparison with the parental EV71:BS strain, the adapted strains accumulated multiple adaptive mutations in the genome resulting in amino acid substitutions, most notably in the capsid-encoding region (P1) and viral RNA-dependent RNA polymerase (3D). Two mutations, E167D and L169F, were mapped to the VP1 canyon that binds the SCARB2 receptor on host cells. Another two mutations, S135T and K140I, were located in the VP2 neutralization epitope spanning amino acids 136–150. This is the first report of human EV71 with the ability to productively infect rodent cell lines *in vitro*.

## Introduction

Enterovirus 71 (EV71) is a small non-enveloped virus approximately 30 nm in diameter. The viral capsid exhibits icosahedral symmetry and is comprised of 60 identical units (protomers), with each consisting of four viral structural proteins VP1–VP4. The capsid surrounds a core of a single-stranded positive-sense RNA genome of 7,450 nucleotides (nt) long. The genome contains a single open reading frame which encodes a polyprotein of 2193 amino acids (aa) and is flanked by a long 5′ untranslated region (UTR) of 745 nt and a shorter 3′ UTR of 85 nt with a poly-A tract of variable length at its 3′ terminus. The polyprotein is divided into three regions, *i.e.* P1, P2 and P3. P1 encodes four viral structural proteins 1A-1D (VP4, VP2, VP3 and VP1); P2 and P3 encode seven non-structural proteins 2A-2C and 3A-3D [1]–[3].

EV71 causes an array of clinical diseases including hand, foot and mouth disease (HFMD), aseptic meningitis, encephalitis and poliomyelitis-like paralysis mainly in infants and young children [4], [5]. The virus was first isolated from a child with acute encephalitis in California, USA in 1969, and subsequently characterized as a new serotype of the genus *Enterovirus* in 1974 [6]. Outbreaks of HFMD with or without neurologic complications and deaths were reported in various parts of the world [7]–[20]. Since 1997, EV71 infections have been a major public health burden and of constant epidemiologic concern in the Asia-Pacific Region. An HFMD outbreak due to highly neurovirulent EV71 emerged in Malaysia resulting in 48 deaths in 1997 [21], [22], followed by a larger outbreak that occurred in Taiwan in 1998 with more than 129,000 cases of HFMD, 405 severe infections and 78 deaths due to acute brainstem encephalomyelitis with neurogenic cardiac failure and pulmonary edema [23]–[26]. In People’s Republic of China, 488,955 HFMD cases with 126 deaths were recorded in 2008 [27] and increased to 1,155,525 cases with 353 fatalities in 2009 [28]. In 2010, China experienced the largest ever HFMD outbreak with more than 1.7 million cases, 27,000 patients with severe neurologic complications, and 905 deaths [29].

Similar to other human enteroviruses, EV71 is unable to infect animals other than humans, although rhesus and cynomolgous monkeys can be experimentally infected [30]–[32]. Understanding its pathogenesis and development of specific therapeutics against the virus are hampered by the lack of suitable small animal models, because EV71 is unable to naturally infect small rodents. Attempts to establish mouse models of EV71 infection and disease have been made, mostly through virus adaptation by serial passages in young suckling mice [33]–[39]. Although some models were able to recapitulate symptoms of clinical illness, none has been reported to cause disease in immune-competent mice aged 2 weeks old or older. Moreover, clinical features of disease and pathology of EV71 infections in humans and experimental monkeys could not be replicated in mice, with the exception of the immunocompromised interferon receptor-deficient AG129 mice [35].

RNA viruses, by virtue of their error-prone replication and high mutation rates [40]–[42], replicate as a swarm of related variant sequences known as *quasispecies* [43], [44]. It is comprised of a master species exhibiting the highest fitness in a certain environment, and of a mutant spectrum composed of a collection of closely related mutant sequences with a certain probability distribution [44], [45]. These endow RNA viruses with genome plasticity, which is reflected in their ability to quickly adapt to changing environments. Despite these endowments, there has been no report of human EV71 that possesses the ability to productively infect rodent-derived cell lines, until now. This manuscript details the phenotypic and genotypic characteristics of two selected mouse cell line (NIH/3T3)-adapted EV71 strains (EV71:TLLm and EV71:TLLmv), which have gained the ability to cause productive infection in cultured rodent cells due to adaptive mutations in the viral genomes. This is the first report of human-derived EV71 that have been successfully adapted to productively infect cell lines of mouse, hamster, and rat origin. EV71:TLLm and EV71:TLLmv represent novel EV71 strains with unique genomic sequences, which can be further applied in developing mouse models of EV71 infection.

## Results

### Primate Cell Lines but not Rodent Cell Lines were Permissible to Infection by EV71:BS

**Figure 1:**
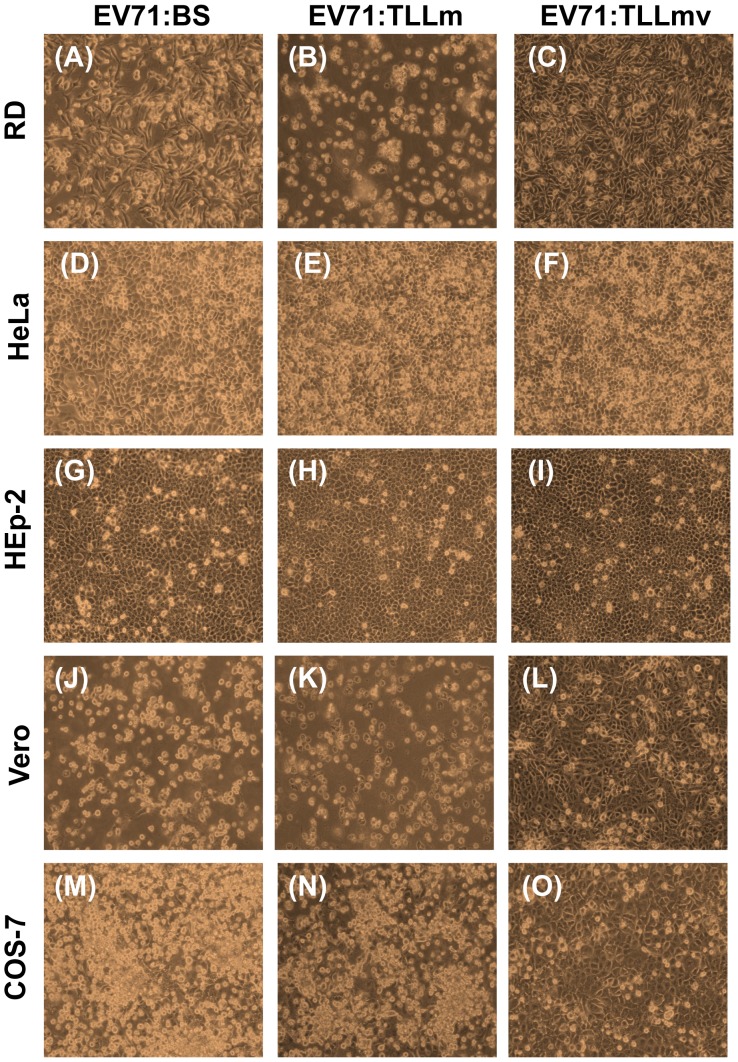
Cytopathic Effects (CPE) observed following virus infection of various primate cell lines. Primate cells: RD cells (A–C), HeLa cells (D–F), HEp-2 cells (G–I), Vero cells (J–L), and COS-7 cells (M–O) infected with 1 MOI of either EV71:BS (A, D, G, J, M), EV71:TLLm (B, E, H, K, N), or EV71:TLLmv (C, F, I, L, O) virus were observed at 48 hpi for cytopathic effects or death of the cell monolayer. Images are representative of results in three independent experiments.

**Figure 2:**
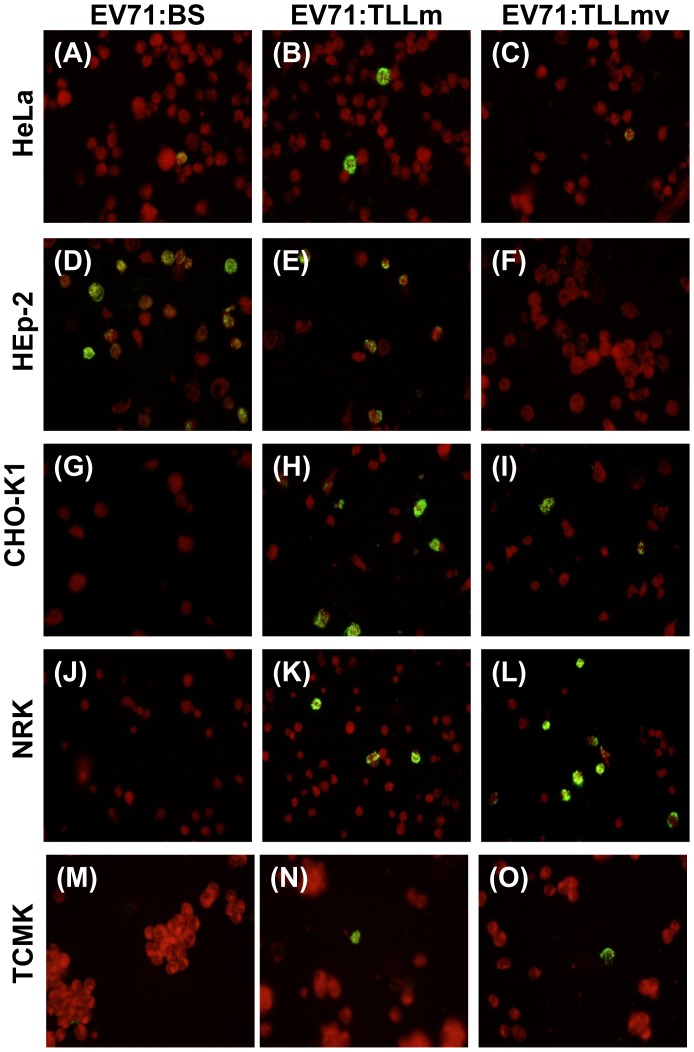
Virus antigen detection in cell lines infected with EV71:BS, EV71:TLLm and EV71:TLLmv. Overnight seeded mammalian cell lines: COS-7 (A–C); HeLa (D–F), HEp-2 (G–I), CHO-K1 (J–L), NRK (M–O), and TCMK (P–R), were infected with 1 MOI of respective virus. Cells were harvested at 48 hpi, coated onto Teflon slides and fixed in cold acetone. Cells were probed with pan-enterovirus antibody and stained with FITC-conjugated anti-mouse IgG. Images are representative of two independent experiments.

**Figure 3:**
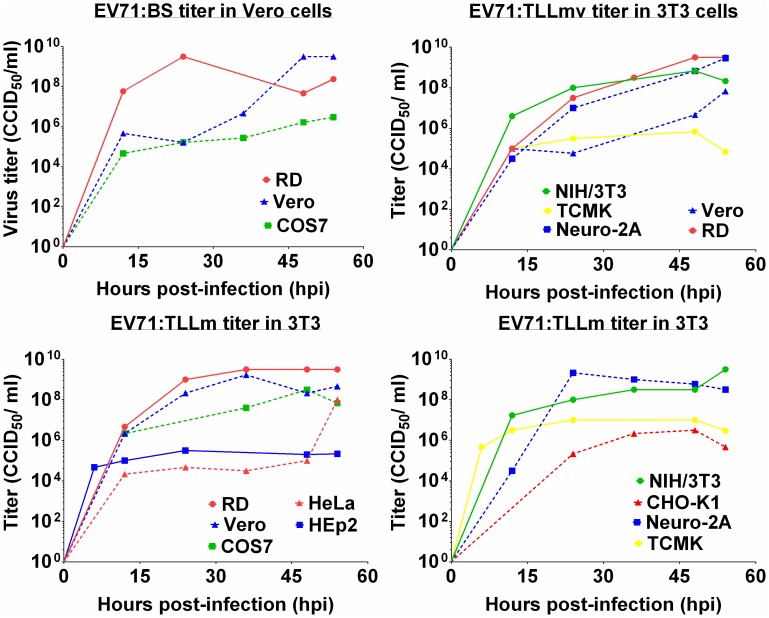
Growth kinetics of EV71:BS, EV71:TLLm, and EV71:TLLmv determined in NIH/3T3 and Vero cells. Supernatants from various mammalian cells infected with 1 MOI of respective virus were harvested at various time points and subjected to titration and enumerated using the Reed and Muench method. (A) EV71:BS virus titer determined in Vero cells. (B) EV71:TLLmv virus titer determined in NIH/3T3 cells. (C, D) EV71:TLLm virus titer determined in NIH/3T3 cells. Growth curves from cell lines that did not exhibit productive infection are not shown.

All primate cell lines used in this study were permissible to infection by EV71:BS virus. Human RD cells, as well as monkey Vero and COS-7 cells, exhibited full lytic cytopathic effects (CPE) within 48 hours post-infection (hpi) (Figure 1A, 1J, 1 M), and viral antigens were detected in fixed infected cells (Figure 2A; data not shown for RD and Vero cells). Growth kinetic curves of the virus harvested from supernatant of various infected cell lines confirmed productive infection in RD, Vero, and COS-7 cells (Figure 3A). Virus harvested from RD and Vero cells reached an endpoint titer of 3×10^9^ CCID_50_/ml, while virus titer from COS-7 was 10^6^ CCID_50_/ml (Figure 3A). EV71:BS did not induce full CPE in HeLa and Hep-2 cells (Figure 1 D, 1G), and the resulting viral titer was not measurable within the assay cut-off limit. However, viral antigen was detected by indirect immunofluorescent staining in both HeLa cells (Figure 2D) and Hep-2 cells (Figure 2G) indicating successful virus entry into the cells but inefficient or defective replication may have resulted in immeasurable virus titer.

**Figure 4:**
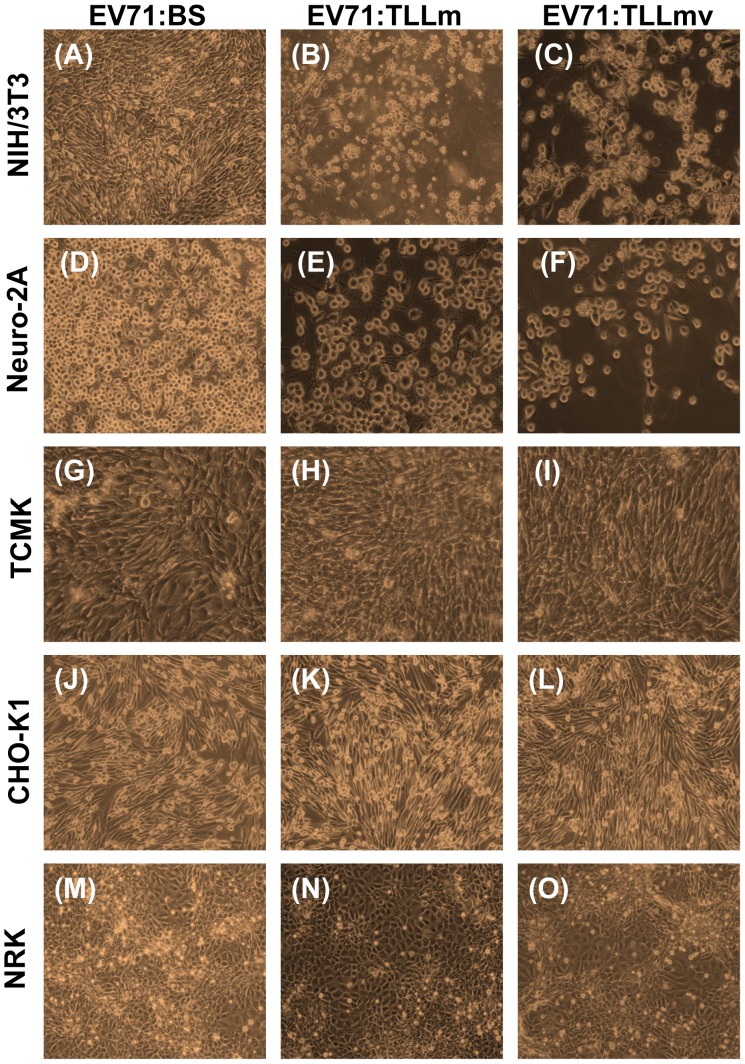
Cytopathic Effects (CPE) observed following virus infection of various rodent cell lines. Rodent cells: NIH/3T3 cells (A–C), Neuro-2A cells (D–F), TCMK cells (G–I), CHO-K1 cells (J–L), and NRK cells (M–O) infected with 1 MOI of either EV71:BS (A, D, G, J, M), EV71:TLLm (B, E, H, K, N), or EV71:TLLmv (C, F, I, L, O) viruses were observed at 48 hpi for cytopathic effects or death of the cell monolayer. Images are representative of results from three independent experiments.

All the rodent cell lines tested were determined to be non-permissive to EV71:BS infection. Lytic CPE of cells was absent following infection (Figure 4A, D, G, J, M), virus titer from supernatants was not measurable, and viral antigens could not be detected in inoculated cells (Figure 2J, M, P; Figure S4A, C, E).

### The Mouse Cell (NIH/3T3)-adapted EV71:TLLm Virus Productively Infected both Primate and Rodent Cell Lines

EV71:TLLm was derived following serial passage of EV71:BS in NIH/3T3 mouse cell line for a minimum of 60 cycles. All primate and rodent cell lines tested, with the exception of NRK cells, were permissible to productive infection by EV71:TLLm. Full CPE was observed in RD, Vero, and COS-7 (Figure 1B, K, N), as well as NIH/3T3 and Neuro-2A cells (Figure 4B, E) at 48 hpi. High virus titer was measurable in supernatants harvested from all infected cell lines except NRK (Figure 3C, 3D), and the infected cells were tested positive for viral antigen by indirect immunofluorescent assay (Figure 2B, E, H, K, Q). Full CPE and measurable viral titer were not observed in NRK cells (Figure 4N, 3D), but viral antigens could be detected (Figure 2N), indicating successful virus entry into NRK cells by EV71:TLLm but inefficient virus replication could have resulted in non-measurable virus titer.

### The Mouse Cell (NIH/3T3)-adapted EV71:TLLmv Virus was able to Productively Infect Rodent Cell Lines but not All Primate Cell Lines

The EV71:TLLmv virus strain was derived from further passage of EV71:TLLm in NIH/3T3 cells for another 40 cycles. EV71:TLLmv caused lytic CPE in fewer number of cell lines - RD, Vero, NIH/3T3, Neuro-2A, and TCMK cells (Figure 3B), and full CPE was only observed in RD, NIH/3T3, and Neuro-2A cells (Figure 1C; 4C, F). TCMK, CHO-K1 and NRK cells were also noted to be permissible to infection without progressing to full CPE (Figure 4I, L, O), as shown by positive viral antigen detection in the infected cells (Figure 2L, O, R).

On the other hand, the primate cell lines HeLa, Hep-2, and COS-7 were observed to be non-permissible to EV71:TLLmv infection, as shown by the absence of CPE (Figure 1F, 1I, 1O), immeasurable virus titers (Figure 3B), and negative viral antigen detection (Figure 2C, F, I).

### EV71:TLLmv Virus Exhibited a Higher Degree of Adaptation to NIH/3T3 Cells, While EV71:TLLm was More Adapted to Replicate in Vero Cells

**Figure 5:**
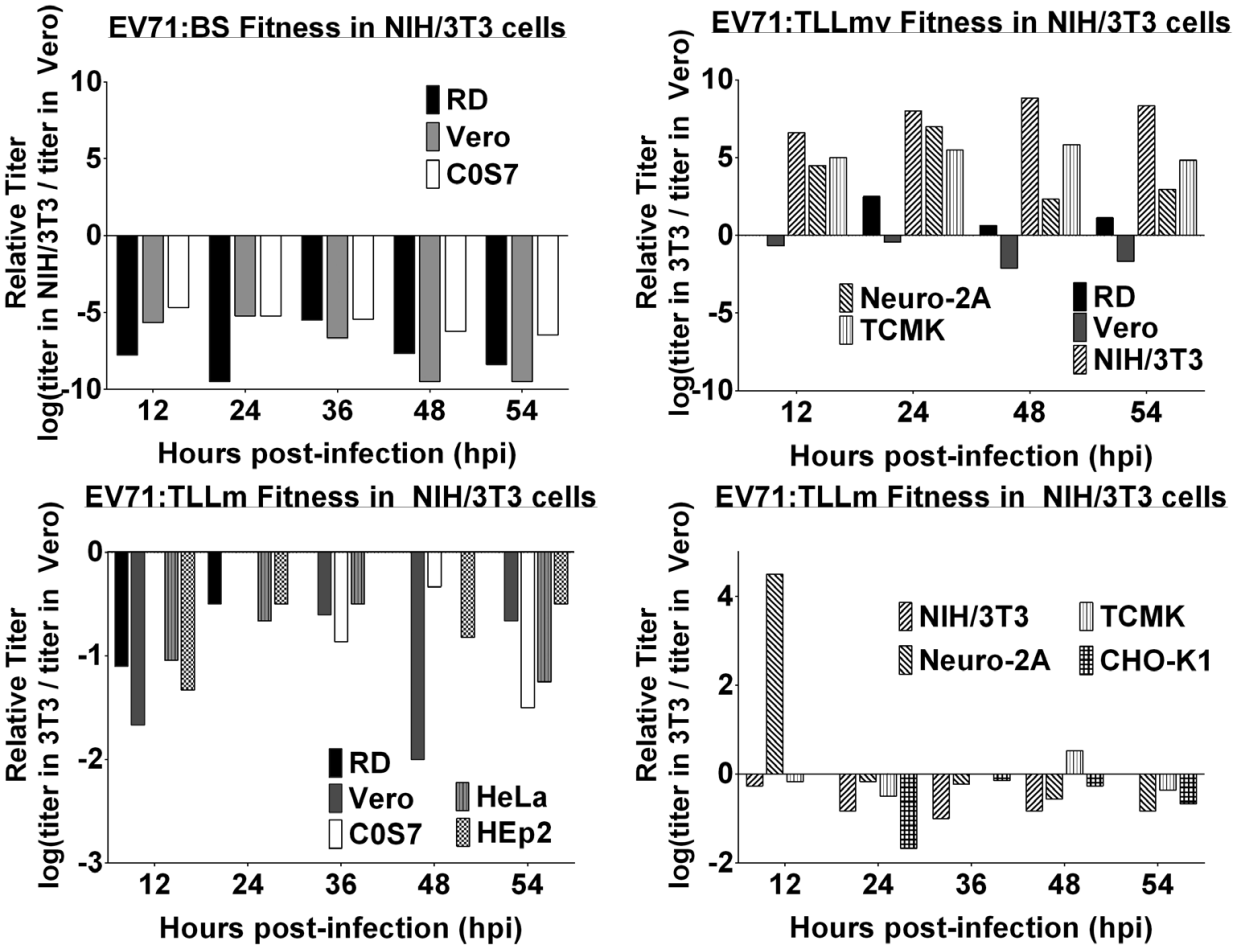
Virus fitness assessment of EV71:BS, EV71:TLLm, and EV71:TLLmv in NIH/3T3 determined by the titer ratio. Virus titer determined separately in NIH/3T3 and Vero cells were used to calculate the virus fitness as log[(titer in NIH/3T3 cells)/(titer in Vero cells)]. Virus fitness of (A) EV71:BS, (B) Ev71:TLLmv, and (C, D) EV71:TLLm were calculated from the virus titer values shown in Figure 3. Virus fitness assays obtained from cell lines that did not exhibit productive infection are not shown.

The amount of viable virus in supernatants harvested from infected cells at various time points was determined by enumerating the virus titer in both Vero and NIH/3T3 cells. The relative reproductive ratio (*RRR*), calculated by taking the ratio of virus titer values assayed in NIH/3T3 to the titer values assayed in Vero, was used as a surrogate measure of the degree of virus adaptation to NIH/3T3 cells. The parental EV71:BS virus displayed highly negative *RRR* values for RD, Vero, and COS-7 (Figure 5A), indicating that the virus titer assayed in Vero cells far exceeds the titer assayed in NIH/3T3 cells. The relative reproductive ratio values for other cell lines could not be determined since the virus titers could not be measured. On the other hand, EV71:TLLmv virus exhibited positive *RRR* values, with the exception of virus propagated in Vero cells (Figure 5B). The positive *RRR* values were indicative of more efficient replication, and therefore higher titer values, in NIH/3T3 cells compared to Vero cells. The negative *RRR* value determined for EV71:TLLmv harvested from Vero cells was consistent with the observed slow growth kinetics (Figure 3B) and lower virus titer. EV71:TLLm exhibited negative *RRR* values (Figure 5C, D), although of lesser degree than the *RRR* values for EV71:BS. This suggested that although EV71:TLLm could productively infect a few rodent cell lines, it was still more adapted to replicate in Vero than NIH/3T3 cells.

### EV71:TLLm Exhibited Better Adaptability to Changing Temperatures than EV71:TLLmv

Vero and NIH/3T3 cells infected with the parental EV71:BS and the derived NIH/3T3-adapted EV71:TLLm and EV71:TLLmv strains were incubated at various temperatures –30°C, 37°C, and 39°C, to determine virus adaptability to temperature variation or changes. EV71:BS displayed the most limited adaptability, with full CPE observed only in Vero cells incubated at 37°C (Figure S2B; Table 1). EV71:TLLmv displayed moderate adaptability, based on the observed full CPE induction in Vero cells at 37°C (Figure S2B) and in NIH/3T3 cells in both 37°C and 39°C (Figure S2A, S3A; Table 1). EV71:TLLm, on the other hand, displayed the greatest adaptability, where it induced full CPE in Vero cells for all incubation temperatures (Figure S1B, S2B, S3B) though only at 37°C in NIH/3T3 cells (Figure S2A; Table 1).

**Table 1:** Assessment of virus adaptability of EV71:BS, EV71:TLLm and EV71:TLLmv grown in NIH/3T3 and Vero cells to various incubation temperatures.

	Induction of full cytopathic effect (CPE) within 48 hours following infection 1_					
Incubation Temperature (°C)	EV71:BS		EV71:TLLm		EV71:TLLmv	
	Vero	NIH/3T3	Vero	NIH/3T3	Vero	NIH/3T3
**30**	–2_	–	+	(30%) 3	–	–
**37**	+3_	–	+	+	+	+
**39**	(20%) 4_	–	+	(<10%) 3	–	+

1 ^_^Infected cells were observed daily for signs of lytic cell death and time of appearance of full CPE.

2 ^_^indicates absence of full CPE in infected cells.

3 ^_^indicates observation of full CPE.

4 ^_^indicates absence of full CPE, while number in parentheses indicates the maximum degree of CPE observed in cells.

### The Viral Genomes of EV71:TLLm and EV71:TLLmv Accumulated Multiple Missense Mutations as a Result of Adaptation to NIH/3T3 Cells

Viral RNA of EV71:BS, EV71:TLLm, EV71:TLLmv were subjected to Sanger sequencing to determine the consensus genome sequence and identify possible adaptive mutations arising from the adaptation process in NIH/3T3 cells. The consensus sequences of the genomes representing dominant population of the quasi-species have been deposited in the GenBank, NCBI (National Center for Biotechnology Information). Alignment of the full genome sequences of EV71:TLLm (GenBank Accession No. KF514879) against EV71:BS (GenBank Accession No. KF514878) revealed 60 nucleotide mutations, 21 of which resulted in amino acid substitutions (Table 2). On the other hand, 83 mutations with 36 amino acid substitutions, were noted between the genomes of EV71:TLLmv (Genbank Accession No. KF514880) and EV71:BS. Majority of the missense mutations were located in the P1 (capsid protein genes) region (Table 2), particularly within the VP1 protein gene (Table 3).

**Table 2:** Nucleotide and amino acid changes in the genomes of EV71:TLLm and EV71:TLLmv compared to EV71:BS.

	EV71:BS vs EV71:TLLm		EV71:BS vs EV71:TLLmv	
Genomic region	No. nucleotide changes	No. amino acid changes	No. nucleotide changes	No. amino acid changes
5′ UTR 5_	11	n/a	11	n/a
	1-bp INS 6_		4-bp INS^18^	
			20-bp DEL 7_	
P1	22	12	39	22
P2	11	2	11	2
P3	16	7	22	12
3′ UTR 8_	0	n/a	0	n/a
**total**	60	21	83	36

5 ^_^5′UTR – 5′ Untranslated Region.

6 ^_^INS – Insertion.

7 ^_^DEL – Deletion.

8 ^_^3′UTR – 3′ Untranslated Region.

**Table 3:** Adaptive mutations observed in the 5′UTR and P1 regions of EV71:TLLm and EV71:TLLmv viral genomes.

	EV71:BS vs EV71:TLLm				EV71:BS vs EV71:TLLmv		
		Amino acid changes				Amino acid changes	
Genome region	Nucleotide changes	Polyprotein *^9_^*	Mature protein *^10—^*	Genome region	Nucleotide changes	Polyprotein^6^	Mature protein^7^
**Cloverleaf**				**Cloverleaf**	C 140 G		
**IRES ** ***^11—^***	A 141 G			**IRES**	A 141 C		
	G 195 C				T 209 C		
	T 209 C				G 258 A		
	G 258 A				C 370 T		
	C 370 T	Not applicable			G 448 A	Not applicable	
	G 448 A				A 502 C		
	T 502 C				G 675 T		
	C 709 T				T 677 C		
	A 671 T				C 687 T		
	G 675 T				C 709 T		
	T 678 INS *^10—^*				678–681 INS		
					726–745 DEL *^13—^*		
**VP4**	A 809 G	E 21 G	E 10 G	**VP4**	A 809 G	E 21 G	E 10 G
**VP2**	G 1359 A	V 204 I	V 126 I	**VP2**	G 1359 A	V 204 I	V 126 I
	G 1385 C	S 213 T	S 135 T		G 1385 C	S 213 T	S 135 T
	A 1400 T	K 218 I	K 140 I		A 1400 T	K 218 I	K 140 I
					T 1428 C	E 228 P	E 150 P
					G 1429 C	E 228 P	E 150 P
**VP3**					G 1900 C	A 385 P	A 62 P
					A 2287 G	T 514 A	T 191 A
					A 2421 G	I 558 M	I 235 M
**VP1**	T 2462 C	V 572 A	V 7 A	**VP1**	T 2462 C	V 572 A	V 7 A
	C 2725 T	I 660 F	I 95 F		C 2530 A	Q 595 K	Q 30 K
	A 2734 G	K 663 E	K 98 E		A 2719 G	I 658 V	I 93 V
	A 2752 G	N 669 D	N 104 D		T 2724 A	D 659 E	D 94 E
	A 2876 G	E 710 A	E 145 A		C 2725 T	I 660 F	I 95 F
	A 2943 T	E 732 D	E 167 D		A 2734 G	K 663 E	K 98 E
	C 2947 T	L 734 F	L 169 F		A 2752 G		
	C 3165 T	S 806 L	S 241 L		A 2753 G	N 669 G	N 104 G
					A 2876 G	E 710 A	E 145 A
					A 2943 T	E 732 D	E 167 D
					C 2947 T	L 734 F	L 169 F
					C 3165 T	S 806 L	S 241 L
					T 3175 C	Y 810 H	Y 245 H
					G 3184 A	V 813 I	V 248 I
					G 3319 T	A 858 S	A 293 S

9 ^—^Numbering of amino acids in the uncleaved polyprotein prior to maturation.

10 ^—^Numbering of amino acids in the mature protein.

11 ^—^IRES - Internal Ribosome Entry Site.

12 ^—^INS – Insertion.

13 ^—^DEL – Deletion.

Amino acid changes were also observed within the P2 and P3 regions, most notably in the RNA-dependent RNA polymerase (3D). EV71:TLLm acquired four amino acid changes, mostly in the palm and thumb domains of the enzyme (Table 4). EV71:TLLmv, on the other hand, accumulated eight amino acid changes, mostly also in the palm and thumb domains of the polymerase. Nucleotide mutations were also observed in the 5′ untranslated region (UTR) of the genome. Apart from base changes, a 1-base insertion was found in EV71:TLLm, while a 4-bp insertion and a 20-bp deletion were observed in EV71:TLLmv 5′UTR (Tables 2 and 3). On the other hand, no amino acid substitutions were observed in the VP3 and 3A regions of EV71:TLLm, as well as in the 3′UTR of both EV71:TLLm and EV71:TLLmv.

**Table 4:** Adaptive mutations observed in the P2 and P3 regions of EV71:TLLm and EV71:TLLmv viral genomes.

	EV71:BS vs EV71:TLLm 14—				EV71:BS vs EV71:TLLmv^11^		
		Amino acid changes				Amino acid changes	
Genome region	Nucleotide changes	Polyprotein 15—	Mature protein 16—	Genome region	Nucleotide changes	Polyprotein	Mature protein
**2A**	G 3722 A	G 992 E	G 130 E	**2A**	G 3722 A	G 992 E	G 130 E
**2C**	A 4366 G	T 1207 A	T 96 A	**2C**	A 4366 G	T 1207 A	T 96 A
**3A**					C 5117 T	A 1457 V	A 17 V
**3B**	T 5357 C	L 1537 P	L 11 P	**3B**	T 5357 C	L 1537 P	L 11 P
**3C**	A 5557 G	I 1604 V	I 56 V	**3C**	A 5557 G	I 1604 V	I 56 V
	A 5569 G	I 1608 V	I 60 V		A 5569 G	I 1608 V	I 60 V
**3D**	A 6211 G	N 1822 D	N 91 D	**3D**	A 6070 T	T 1775 S	T 44 S 17—
	T 6835 A	S 2030 T	S 299 T^4^		T 6137 C	V 1797 A	V 66 A^3^
	A 7128 G	R 2127 S	R 396 S^5^		A 6211 G	N 1822 D	N 91 D
	T 7247 C	V 2167 A	V 436 A^5^		C 6404 G	S 1886 C	S 155 C
					T 6592 G	W 1949 G	W 218 G 18—
					T 6835 A	S 2030 T	S 299 T^4^
					A 7128 G	R 2127 S	R 396 S 19—
					T 7247 C	V 2167 A	V 436 A^5^

14 ^—^Mutations are based on alignment with the reference EV71:BS genome.

15 ^—^Numbering of amino acids in the uncleaved polyprotein prior to maturation.

16 ^—^Numbering of amino acids in the mature protein.

17 ^—^Mutation located in the Ring finger domain of the RNA-dependent RNA Polymerase.

18 ^—^Mutation located in the Palm domain of the RNA-dependent RNA Polymerase.

19 ^—^Mutation located in the Thumb domain of the RNA-dependent RNA Polymerase.

### Transfection of EV71:BS Viral RNA into Murine Cells Resulted in Productive Infection but the Virus Progeny could not Re-infect the Same Mouse Cells

**Figure 6:**
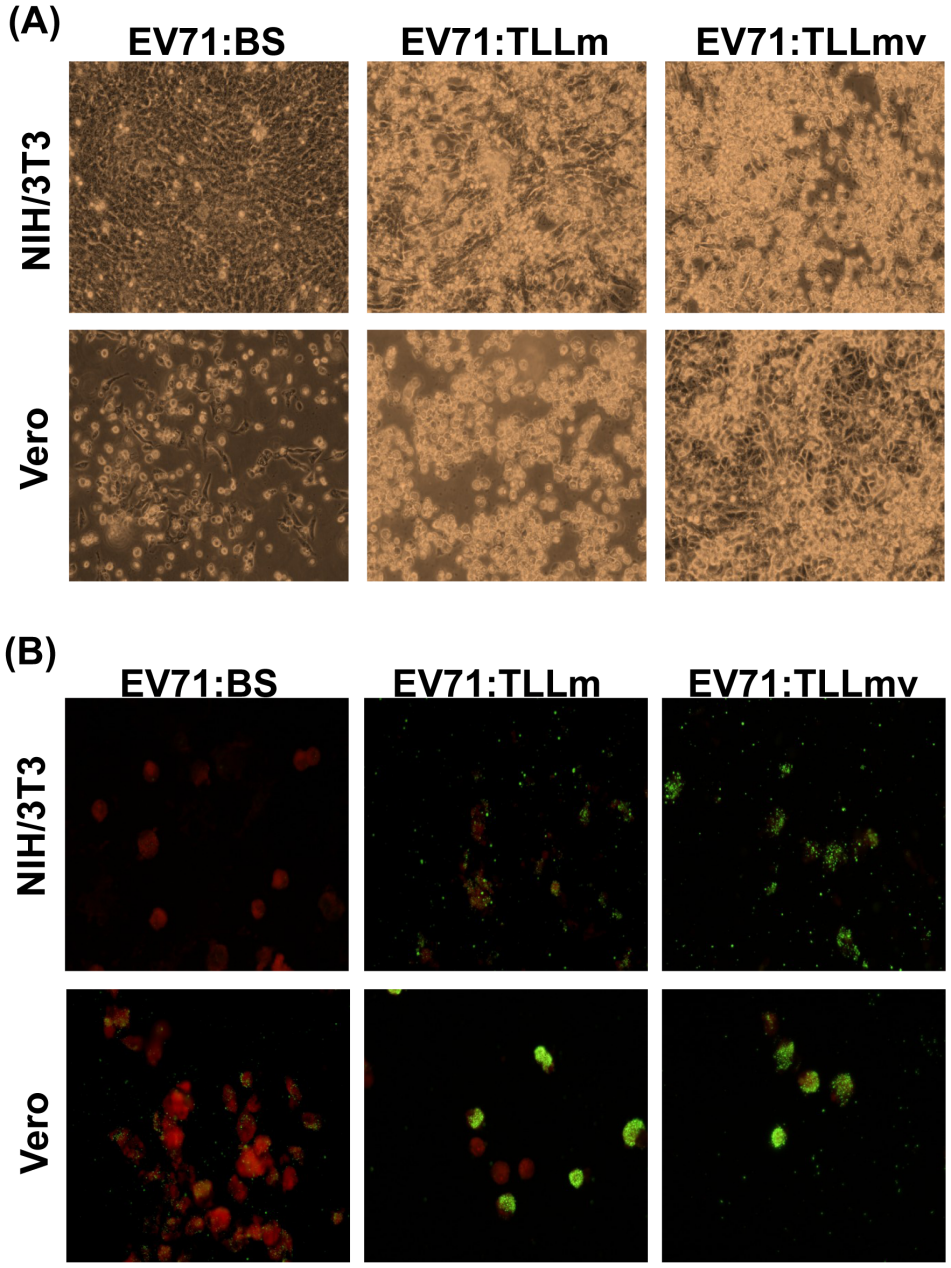
Transfection of NIH/3T3 with EV71:BS viral RNA induces productive infection. Overnight seeded NIH/3T3 and Vero cells were inoculated with virus supernatant harvested from NIH/3T3 cells previously transfected viral RNA extracted from EV71:BS, EV71:TLLm, and EV71:TLLmv. (A) Cells were imaged using inverted light microscope at 24 hpi to observe induced CPE. (B) Cells were harvested at 7 dpi, coated onto Teflon slides, probed with pan-enterovirus antibody, and stained with anti-mouse FITC-conjugated antibody.

Vero and NIH/3T3 cells transfected with viral RNA exhibited full CPE at 7 days post-transfection (dpt) (data not shown). Viral antigens were detected in NIH/3T3 cells transfected with viral RNA of EV71:BS (Figure S4B), but not in NIH/3T3 cells subjected to infection with the virus (Figure S4A). Virus supernatants re-inoculated onto fresh Vero and NIH/3T3 cells resulted in productive infection (100% CPE) only in Vero but not in NIH/3T3 cells (Figure 6A), and viral antigen detection confirmed infection in Vero cells, but not NIH/3T3 (Figure 6B).

## Discussion

Sequential passage of the human EV71 isolate (EV71:BS) generated virus strains that gained the ability to infect *in vitro* cultured rodent cell lines. The mouse adherent fibroblast cell line NIH/3T3, which was derived from the NIH/Swiss mouse embryo [46], was used to adapt the human EV71 strain to infect rodent cells. We report two such NIH/3T3-adapted strains - EV71:TLLm and EV71:TLLmv, where EV71:TLLm represents the early stage (passage number 60) and EV71:TLLmv represents the late stage (passage number 100) of the adaptation process. Based on the appearance of virus-induced CPE, measurement of high titer values, and positive detection of viral antigen through immunostaining, we categorized the virus-induced infection in cells as either productive or non-productive. Productive infection exhibits positive viral antigen detection as well as high virus titers, regardless of observation of CPE. On the other hand, non-productive infection is characterized by immeasurable virus titer at cut-off assay limit despite viral antigen detection and/or observation of CPE.

Whereas the clinical isolate EV71:BS infects only primate cell lines, EV71:TLLm productively infects both primate and rodent cell lines. It is worth noting that although EV71:TLLm virus has successfully achieved the ability to infect rodent cells, the degree of adaptation to NIH/3T3 cells is less pronounced. The virus titer determined using Vero cells is much higher than that determined in NIH/3T3 cells, as indicated by negative values in relative replication rates (RRR) assay (Figure 5C, D). In addition, EV71:TLLm successfully infects Vero cells leading to full CPE at various incubation temperatures whereas it can only achieve full CPE in infected NIH/3T3 at 37°C (Table 1). Further adaptation in mouse cells, which yielded the EV71:TLLmv virus, resulted in a virus strain that displays a higher degree of adaptation to mouse cells (Figure 5B) but at the cost of narrowing down the spectrum of permissible host cells. EV71:TLLmv does not infect primate cell lines as effectively as mouse cells although it exhibits successful infection leading to full CPE of NIH/3T3 cells incubated at a broader range of temperatures (Table 1). However, compared to the predecessor EV71:TLLm, EV71:TLLmv seemed to have lost the ability to enter and replicate in monkey kidney COS-7 cells, as well as human HeLa and Hep-2 cells (Figure 3B–C; 2B–C, E–F, H–I). It also lost the ability to replicate efficiently within hamster CHO-K1 and rat NRK cells (Figure 3B–D; 2K–L, N–O). These observations indicate that further passage of the virus in NIH/3T3 cells increases the degree of adaptation in mouse cells at the cost of losing infective ability in cell lines of other origin.

Although it is not possible to pinpoint which amino acid substitutions are adaptive to the mouse NIH/3T3 host cell, and this subject is not covered in this manuscript, viral whole genome sequencing may shed light to potential adaptive mechanisms. Most of the amino acid substitutions identified reside in the P1 (capsid) and RNA polymerase (3D) proteins (Table 2, 3), suggesting possible altered virus protein activity in host cell entry and replication. The accumulation of mutations in the P1 region is expected from the acquired ability of EV71:TLLm and EV71:TLLmv strains to infect new host cells. Capsid proteins form the structural context with which the virus initiates interaction with the permissive host cell through the virus receptor, which had been identified recently as Scavenger Receptor Class B Member 2 (SCARB2) [47] and later characterized as the main virus uncoating receptor of EV71 [48] and which is also utilized by some members of Human Enterovirus A (HEV-A) species. The human SCARB2 protein shares approximately 99% sequence identity with that of other primates. On the other hand, mouse SCARB2 protein exhibits 15% sequence dissimilarity compared to the primate protein [49], implying significant structural deviations from primate SCARB2 and perhaps contributing to the recalcitrance of rodent cells to native EV71 infection. It is plausible that adaptive mutations in the virus capsid may render the virus competent to bind the mouse cell receptor and result in successful entry and infection of novel hosts.

Mapping of the capsid protein mutations indicate that majority of the identified amino acid substitutions in the viral P1 region reside in exposed regions of the protein (Figure S5), specifically in the B–C, D–E, E–F, and G-H loops on the surface of VP1. The VP1 residues 150–180 harbour the viral capsid canyon that engages SCARB2 protein. This region centred at Gln-172 contains a major VP1 neutralization epitope at amino acids 163–177 [32]. Both EV71:TLLm and EV71:TLLmv exhibited substitutions E167D and L169F in the E-F loop of the VP1 canyon (Figure S5A–B), loci which have not been previously reported. Other significant amino acid substitutions near the SCARB2 docking site include N104D within the B–C loop and S241L in the G-H loop, which are located within a 20 Å radius from Gln-172. The VP1 S241L mutation, in association with the K244E, had been previously reported arising from mouse passage of a CHO cell line-adapted EV71 [50]. This mutation, in combination with a VP2 K149I, was found to be associated with a non-virulent phenotype in 5-day mouse pups. However, a reverse mutation at VP1 241 from Leu to Ser [51] was reported arising from adaptation in NOD/SCID mouse brain tissues and found to be associated with a mouse virulent phenotype. The VP1 E145A mutation, which is far from the SCARB2 docking site and located in the D–E loop, is another candidate for conferring the ability to infect murine cells. The VP1 145 mutation had been previously reported [34], [37] and a single E145A mutation leads to virulence in NOD/SCID mice [51]. Another mutation in VP1 of a C4 genotype EV71, Q145E, was associated with virulence in 5-day old mice [52]. The mouse cell-adaptive mutations in VP2, particularly those within the neutralizing epitope of residues 136–150 [53], may also contribute to the virus ability to infect rodent cells. Two substitutions in VP2 E-F loop were observed in EV71:TLLm (Figure S6C), while three substitutions were present in EV71:TLLmv (Figure S5D). None of these mutations have been previously described, although a nearby locus at VP2 149 in the E-F loop had been mentioned in the literature [34], [50], [54] and described as an adaptive mutation to passage in PSGL-1 overexpressing cells [55].

To explore the possible role of the P1 region mutations in binding the virus receptor for host cell entry, EV71:BS viral RNA was transfected into murine cells. Direct introduction of EV71:BS RNA into the mouse cell cytoplasm results to productive infection in NIH/3T3 cells, as suggested by the observation of virus-induced CPE and measurable virus titers in the culture supernatant as assayed in Vero cells (Figure S6). Re-inoculation of the virus supernatant onto fresh NIH/3T3 cells, however, fails to induce productive infection (Figure 6A) and no viral antigens were detected (Figure S4H). Similarly, transfection of EV71:BS RNA into Neuro-2A cells, but not direct virus infection, resulted in positive antigen staining in Neuro-2A cells (Figure S4C, S4D) and measurable virus titers (Figure S6). Virus supernatants passaged onto fresh Vero and NIH/3T3 cells resulted to positive antigen staining in Vero (Figure S4I) but not in NIH/3T3 cells (Figure S4J). These data indicate that circumventing the requirement of receptor engagement for host cell entry led to successful infection and production of virus progenies in NIH3T3 and Neuro-2A cells. These data also support that human EV71:BS cannot successfully enter mouse NIH/3T3 and Neuro-2A cells through murine cellular receptor. Moreover, these data suggest that mutations within the P1 region of EV71:TLLm and EV71:TLLmv genomes confer the virus with the ability for efficient receptor engagement, and consequently host cell entry.

Several amino acid mutations were also observed in the P2 and P3 regions, which encode virus proteins that are crucial for virus replication and hijacking the host cell protein translation machinery [56]. These adaptive mutations may function in optimizing EV71 genome replication and translation within mouse cells. The viral 3D protein alone accumulated 8 amino acid substitutions in EV71:TLLmv and 4 in EV71:TLLm, and both strains exhibited 1 mutation each in 3B and 2 mutations each in 3C. Direct inoculation of viral RNA into TCMK cells give insight into the possible role of the adaptive mutations in the nonstructural proteins of the virus. Although TCMK cells have been shown to be permissible to EV71:TLLm and EV71:TLLmv infection (Figure 3B, D; 4Q, R), transfection of EV71:BS viral RNA into TCMK cells did not result to successful infection. Viral antigen signals were not detected in infected and transfected cells (Figure S4E–F), and passage of virus supernatant onto fresh NIH/3T3 and Vero cells did not yield positive viral antigen detection (Figure S4K, L). Moreover, there was no assayable virus titer in both infected and transfected cells (Figure S6). These data suggest that apart from mutations in the capsid region, mutations within the P2 and P3 regions, which are found in EV71:TLLm and EV71:TLLmv, are required to successfully infect TCMK cells. The specific role of these adaptive mutations, which is the focus of ongoing studies, is beyond the scope of this manuscript.

To our knowledge, this is the first report of EV71 strains originally derived from human clinical sample that have successfully gained the ability to productively infect several rodent cell lines following successive passages in a mouse cell line. The relatively high mutation rates during RNA replication results in variant genomes that serve as genetic reservoirs of phenotypic traits for future adaptive potential, and the consequential replication as a quasispecies distribution [57], [58] leads to the dynamic plasticity of RNA viral genomes conferring adaptability to changing environments [59], [60]. Although EV71 infecting suckling mice [34], [37], [38], [51] and other rodents (e.g. gerbils) [39] have been reported previously, none was reported to productively infect *in vitro* cultured rodent cells. This may be due to the high genetic barrier associated with major changes in phenotype and host range [58]. Interestingly, our group is the first to report a large number of adaptive mutations within the consensus full genome sequence of EV71. Whereas previously documented mouse-adapted EV71 reported less than 10 amino acid substitutions in the genome [34], [38], [51], we report 21 in EV71:TLLm and 36 in EV71:TLLmv, still more than the most number of adaptive mutations previously identified [37]. These suggest that few passages of the virus in mouse tissues [34], [36], [37] may not be sufficient to break the genetic barrier and successfully adapt the virus to infect cultured mouse cells. Instead, hundreds of successive passages might be necessary, as is observed in this study.

The mouse cell line (NIH/3T3)-adapted virus strains presented in this study may provide an alternative opportunity in establishing mouse models of EV71 infections in immune-competent mice. Preliminary findings of animal studies demonstrated that BALB/c mice aged 14 days old could be acutely infected with EV71:TLLm (10^6^ CCID_50_ per mouse) via intragastric and intraperitoneal routes (conducted with approval by the Institutional Animal Care and Use Committee). A proportion of the infected pups succumbed to acute illness leading to death within a few days post-infection (unpublished data). This preliminary result seems promising, and further studies are required to ascertain the usefulness of these virus strains in establishing mouse models of EV71 infection, pathogenesis and disease.

## Materials and Methods

### A. Cell Lines and Virus Strains used in the Study

All cell lines used in this study were purchased from the American Tissue Type Culture Collection (ATCC, USA). Studies were performed using various mammalian cell lines; human adenocarcinoma cell lines HeLa (CCL-2) and HEp-2 (CCL-23), and rhabdomyosarcoma RD (CCL-136); African green monkey kidney Vero (CCL-81), and Vervet monkey kidney fibroblast COS-7 (CRL-1651); mouse neuroblastoma Neuro2A (CCL-131), embryonic fibroblast NIH/3T3 (CRL-1658), and kidney epithelial TCMK (CCL-139); hamster ovarian epithelial-like CHO-K1 (CCL-61), and normal rat kidney epithelial NRK (CRL-6509).

The human EV71 BS strain (EV71:BS) was previously isolated from the brainstem of a deceased patient infected with EV71. The virus was passaged in Vero cells for four cycles prior to storage at −80°C until further use. The mouse cell (NIH/3T3)-adapted EV71:TLLm strain was derived from the EV71:BS strain via continuous serial passage (>60 cycles) in mouse NIH/3T3 cells. The EV71:TLLm strain was further passaged (40 cycles) in NIH/3T3 cells to generate the mouse cell-adapted virulent strain (EV71:TLLmv).

### B. Maintenance of Cell Lines and Infection with Virus

All cell lines were grown in Dulbecco’s Modified Eagle’s Medium (DMEM, Gibco, USA) supplemented with 10% (^v^/_v_) of fetal bovine serum (FBS, i-DNA Singapore) and 0.22% (^w^/_v_) sodium bicarbonate (NaHCO_3_, Sigma Aldrich, USA) and incubated at 37°C and 5% CO_2_, unless otherwise stated. All infected cells were incubated in maintenance medium (DMEM supplemented with 1% FBS and 0.22% NaHCO_3_).

Cells (2.5–5.0×10^5^ cells per well) were seeded in tissue culture-treated six-well plates (Nunc, Fisher Scientific) overnight, infected with 500 μl of virus suspension (MOI 1), and incubated at 30°C, 37°C, or 39°C for 2 hours. Cells were washed twice in sterile Phosphate-Buffered Saline (PBS, pH 7.4) solution before addition of fresh maintenance medium (DMEM, 1% FBS). Infected cells were observed daily for appearance of distinct lytic cytopathic effects (CPE).

For virus growth kinetic studies, plates containing the infected cells were frozen at −80°C at various time-points: 0, 6, 12, 24, 36, 48, and 54 hours post-infection (hpi). Plates were subjected to three cycles of freezing and thawing, and lysates were harvested and cleared by vigorous vortexing followed by centrifugation at 1,500×g for 10 minutes. Cleared supernatants were stored in cryovials (Nunc, Fisher Scientific) at −80°C until further use.

For temperature adaptation assays, inoculated Vero and NIH/3T3 cells were incubated at 30°C, 37°C, and 39°C and observed daily for appearance of CPE. Respective culture supernatants were harvested at 48 hpi and stored in cryovials at −80°C until further use.

Various mammalian cell lines, i.e. RD, HeLa, and HEp-2 (human), Vero and COS-7 (monkey), NIH/3T3, Neuro-2A, and TCMK (mouse), CHO-K1 (hamster), and NRK cells (rat), were infected with either parental EV71:BS or derived NIH/3T3-adapted EV71 strains at MOI (multiplicity of infection) of 1 and incubated at 37°C for 10 days. Cultures were observed daily for appearance of CPE.

### C. Determination of Virus Titer and Relative Replication Rates (RRR)

Virus supernatants were subjected to endpoint titration and assayed in both NIH/3T3 and Vero cells. The virus titer was enumerated using the Reed and Muench method [61] and the Reed and Muench calculator [62]. Briefly, NIH/3T3 (1×10^4^cells per well) and Vero cells (4×10^3^ cells per well) were seeded overnight in a 96-well plate. Frozen virus thawed to room temperature were diluted (10^−1^) in sterile 1% aqueous sodium deoxycholate (Sigma Aldrich, USA), and vigorously mixed for 15 minutes to disaggregate virus. Disaggregated virus was subjected to ten-fold serial dilution in maintenance medium, and 100 μl diluted virus from 10^−3^ dilution onwards was added onto each well of cells. Plates were incubated at 37°C and observed daily under inverted light microscopy for the appearance of distinct CPE. Virus titer was reported as 50% cell culture-infectious doses per volume (CCID_50_/ml).

To assess the degree of adaptation of EV71:TLLm and EV71:TLLmv in NIH/3T3 cells, virus supernatants harvested from previously infected primate and rodent cell lines were subjected to virus titer assay using both NIH/3T3 and Vero cells. The titer ratio used to measure *relative replication rates* (RRR) in NIH/3T3 and Vero cells was calculated using the following formula:
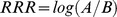
where *A* is the virus titer assayed in NIH/3T3 cells, and *B* is the virus titer assayed in Vero cells.

### D. Virus Antigen Detection by Immunofluorescence Assay

For infected cells that did not exhibit significant CPE, immunofluorescence (IF) staining was performed to verify infection. Cells were trypsinized at 72 hpi, washed twice in sterile PBS, and coated onto Teflon slides (Erie, USA). Slides were air-dried inside the biosafety cabinet and UV-treated for 15 minutes to inactivate live virus prior to fixation in cold acetone at 4°C for 10 minutes. Slides were probed with pan-Enterovirus antibody (Merck Millipore, USA) and subsequently with FITC-conjugated mouse IgG (DAKO Cytomation, Denmark) mixed with 0.01% (^w^/_v_) Evan’s blue counter stain.

### E. Transfection of Cells with EV71 Viral RNA

Vero and NIH/3T3 cells (3×10^4^ cells per well) seeded overnight in 24-well plates were transfected with viral RNA extracted from 4×10^6^ CCID_50_ virus using Lipofectamine 2000 (Life technologies, USA) following the manufacturer’s protocol. RNA from EV71:BS, EV71:TLLm, and EV71:TLLmv was extracted using Viral RNA kit (Qiagen, Germany) and incubated with Lipofectamine 2000 on cells for 6 hours at 37°C. Transfected cells were observed daily for appearance of CPE. At 7 dpi, supernatant was harvested from infected cells and passaged onto freshly seeded Vero and NIH/3T3 cells. Cells were observed daily for appearance of CPE, and at 7 dpi, cells were trypsinized and processed for immunofluorescence viral antigen detection.

### F. Full Genome Sequencing and Genetic Mapping of EV71 Strains

Viral RNA of EV71:BS, EV71:TLLm, and EV71:TLLmv strains was extracted using Viral RNA kit (Qiagen, Germany) and reverse-transcribed using Superscript II (SII-RT, Life Technologies, USA). The cDNA obtained was amplified with GoTaq Green (Promega, USA) and degenerate EV71 primers (primers’ sequences are available upon request). The amplicon was purified using PCR clean up kit (Geneaid Biotech, Taiwan) and cloned into pZero2 vector (Lifetech, USA). Clones were selected from Kanamycin plates, inoculated into LB broth (50 μg/ml Kanamycin) and allowed to grow overnight at 37°C for plasmid extraction with QiaSpin Miniprep kit (Qiagen, Germany). Plasmids were subsequently sequenced by the BigDye terminator method (Applied Biosystems, USA) using the same primers. The 500-bp fragment sequences obtained were aligned using BioEdit v7.0.9.0 [63] against the whole genome sequence of an EV71 Singapore isolate 3799-SIN-98 (GenBank accession no. DQ341354.1) to reconstruct the full genome sequences of EV71:BS, EV71:TLLm, and EV71:TLLmv. Molecular modelling of the protomers of EV71:TLLm, and EV71:TLLmv was performed using Deepview/Swiss pdbviewer ver. 3.7 (http://expasy.org/spdbv/) and the SWISS-MODEL server [64], [65].

## Supporting Information

Figure S1 **Virus fitness assessment of EV71:BS, EV71:TLLm, and EV71:TLLmv in NIH/3T3 and Vero cells at 30**°**C.** Overnight seeded (A) NIH/3T3 and (B) Vero cells infected with EV71:BS (a, d, g), EV71:TLLm (b, e, h), or EV71:TLLmv (c, f, i) were incubated at 30°C and observed under the light microscope with phase-contrast at 24 hpi (a–c), 48 hpi (d–f), and 72 hpi (g–i). Images taken are representative of two independent experiments.
(TIF)

Figure S2 **Virus fitness assessment of EV71:BS, EV71:TLLm, and EV71:TLLmv in NIH/3T3 and Vero cells at 37**°**C.** Overnight seeded (A) NIH/3T3 and (B) Vero cells infected with EV71:BS (a, d, g), EV71:TLLm (b, e, h), or EV71:TLLmv (c, f, i) were incubated at 37°C and observed under the light microscope with phase-contrast at 24 hpi (a–c), 48 hpi (d–f), and 72 hpi (g–i). Images taken are representative of two independent experiments.
(TIF)

Figure S3 **Virus fitness assessment of EV71:BS, EV71:TLLm, and EV71:TLLmv in NIH/3T3 and Vero cells at 39**°**C.** Overnight seeded (A) NIH/3T3 and (B) Vero cells infected with EV71:BS (a, d, g), EV71:TLLm (b, e, h), or EV71:TLLmv (c, f, i) were incubated at 39°C and observed under the light microscope with phase-contrast at 24 hpi (a–c), 48 hpi (d–f), and 72 hpi (g–i). Images taken are representative of two independent experiments.
(TIF)

Figure S4 **Transfection of murine cell lines NIH/3T3, Neuro-2A, and TCMK with EV71:BS viral RNA for evidence of virus replication.** Overnight seeded NIH/3T3, Neuro-2A, and TCMK cells were either infected with 1000 CCID_50_ of EV71:BS virus (A, C, E) or transfected with equivalent amounts of viral RNA (B, D, F). and harvested at 48 hpi for viral antigen detection. Virus in the supernatants were harvested at 7 dpi and passaged onto fresh Vero (G, I, K) and NIH/3T3 cells (H, J, L). Cells were harvested and stained for viral antigens at 48 hpi.
(TIF)

Figure S5 **Localization in VP1 and VP2 of adaptive mutations in the genomes of EV71:TLLm and EV71:TLLmv.** Adaptive mutations observed in the VP1 (A, B) and VP2 (C, D) regions of EV71:TLLm (A, C) and EV71:TLLmv (B, D) were modelled using DeepView/SwissPDBviewer v3.7 and the 3D structure of EV71 capsid P1 region (PDB ID 4AED). The mutations were observed to be mostly localized to the surface-exposed loops of the protein. The B–C loop is shown in red; D–E loop in yellow; E–F loop in orange; and G–H loop in pink.
(TIF)

Figure S6 **Titer ratio in NIH/3T3 cells relative to titer in Vero cells of virus supernatant harvested from cells either transfected with EV71:BS viral RNA or infected with live virus.** Supernatants from NIH/3T3, Neuro-2A, Vero, and TCMK either transfected with viral RNA or infected with live virus were harvested and subjected to virus enumeration by Reed-and Muench method. The ratio of the log(titer) determined in NIH/3T3 cells relative to the titer determined in Vero cells is shown. *3T3-TRANS:* RNA transfected NIH/3T3 cells; *3T3-INF:* virus infected NIH/3T3 cells. Asterisks indicate Student’s t-test with p-value <0.05.
(TIF)
