# Correction: Phenotypic and Genotypic Characteristics of Novel Mouse Cell Line (NIH/3T3)-Adapted Human Enterovirus 71 Strains (EV71:TLLm and EV71:TLLmv)

**DOI:** 10.1371/journal.pone.0112334

**Published:** 2014-10-30

**Authors:** The PLOS ONE Staff

There are errors in Table 3. Please see the corrected Table 3 here.

**Table 3:** Adaptive mutations observed in the 5'UTR and P1 regions of EV71:TLLm and EV71:TLLmv viral genomes.

	EV71:BS vs EV71:TLLm				EV71:BS vs EV71:TLLmv		
		Amino acid changes				Amino acid changes	
Genome region	Nucleotide changes	Polyprotein^1^	Mature protein^2^	Genome region	Nucleotide changes	Polyprotein^6^	Mature protein^7^
**Cloverleaf**				**Cloverleaf**	C 140 G		
**IRES** 3	A 141 G			**IRES**	A 141 C		
	G 195 C				T 209 C		
	T 209 C				G 258 A		
	G 258 A				C 370 T		
	C 370 T	Not applicable			G 448 A	Not applicable	
	G 448 A				A 502 C		
	T 502 C				G 675 T		
	C 709 T				T 677 C		
	A 671 T				C 687 T		
	G 675 T				C 709 T		
	T 678 INS^4^				678-681 INS		
					726-745 DEL^5^		
**VP4**	A 809 G	E 21 G	E 21 G	**VP4**	A 809 G	E 21 G	E 21 G
**VP2**	G 1359 A	V 204 I	V 135 I	**VP2**	G 1359 A	V 204 I	V 135 I
	G 1385 C	S 213 T	S 144 T		G 1385 C	S 213 T	S 144 T
	A 1400 T	K 218 I	K 149 I		A 1400 T	K 218 I	K 149 I
					T 1428 C	E 228 P	E 159 P
					G 1429 C	E 228 P	E 159 P
**VP3**					G 1900 C	A 385 P	A 62 P
					A 2287 G	T 514 A	T 191 A
					A 2421 G	I 558 M	I 235 M
**VP1**	T 2462 C	V 572 A	V 7 A	**VP1**	T 2462 C	V 572 A	V 7 A
	C 2725 T	L 660 F	L 95 F		C 2530 A	Q 595 K	Q 30 K
	A 2734 G	K 663 E	K 98 E		A 2719 G	I 658 V	I 93 V
	A 2752 G	N 669 D	N 104 D		T 2724 A	D 659 E	D 94 E
	A 2876 C	E 710 A	E 145 A		C 2725 T	L 660 F	L 95 F
	A 2943 T	E 732 D	E 167 D		A 2734 G	K 663 E	K 98 E
	C 2947 T	L 734 F	L 169 F		A 2752 G		
	C 3165 T	S 806 L	S 241 L		A 2753 G	N 669 G	N 104 G
					A 2876 C	E 710 A	E 145 A
					A 2943 T	E 732 D	E 167 D
					C 2947 T	L 734 F	L 169 F
					C 3165 T	S 806 L	S 241 L
					T 3175 C	Y 810 H	Y 245 H
					G 3184 A	V 813 I	V 248 I
					G 3319 T	A 858 S	A 293 S
							

1 Numbering of amino acids in the uncleaved polyprotein prior to maturation.

2 Numbering of amino acids in the mature protein.

3 IRES - Internal Ribosome Entry Site.

4 INS – Insertion.

5 DEL – Deletion.

There are errors in Table 4. Please see the corrected Table 4 here.

**Table 4:** Adaptive mutations observed in the P2 and P3 regions of EV71:TLLm and EV71:TLLmv viral genomes.

	EV71:BS vs EV71:TLLm^6^				EV71:BS vs EV71:TLLmv^11^		
		Amino acid changes				Amino acid changes	
Genome region	Nucleotide changes	Polyprotein^7^	Mature protein 8	Genome region	Nucleotide changes	Polyprotein	Mature protein
**2A**	G 3722 A	G 992 E	G 130 E	**2A**	G 3722 A	G 992 E	G 130 E
**2C**	A 4366 G	T 1207 A	T 96 A	**2C**	A 4366 G	T 1207 A	T 96 A
**3A**					C 5117 T	A 1457 V	A 17 V
**3B**	T 5357 C	L 1537 P	L 11 P	**3B**	T 5357 C	L 1537 P	L 11 P
**3C**	A 5557 G	I 1604 V	I 56 V	**3C**	A 5557 G	I 1604 V	I 56 V
	A 5569 G	I 1608 V	I 60 V		A 5569 G	I 1608 V	I 60 V
**3D**	A 6211 G	N 1822 D	N 91 D	**3D**	A 6070 T	T 1775 S	T 44 S^9^
	T 6835 A	S 2030 T	S 299 T^4^		T 6137 C	V 1797 A	V 66 A^3^
	A 7128 T	R 2127 S	R 396 S^5^		A 6211 G	N 1822 D	N 91 D
	T 7247 C	V 2167 A	V 436 A^5^		C 6404 G	S 1886 C	S 155 C
					T 6592 G	W 1949 G	W 218 G^10^
					T 6835 A	S 2030 T	S 299 T^4^
					A 7128 T	R 2127 S	R 396 S^11^
					T 7247 C	V 2167 A	V 436 A^5^

6 Mutations are based on alignment with the reference EV71:BS genome.

7 Numbering of amino acids in the uncleaved polyprotein prior to maturation.

8 Numbering of amino acids in the mature protein.

9 Mutation located in the Ring finger domain of the RNA-dependent RNA Polymerase.

10 Mutation located in the Palm domain of the RNA-dependent RNA Polymerase.

11 Mutation located in the Thumb domain of the RNA-dependent RNA Polymerase.

## References

[1] Victorio CBL, Xu Y, Ng Q, Chow VTK, Chua KB (2014) Phenotypic and Genotypic Characteristics of Novel Mouse Cell Line (NIH/3T3)-Adapted Human Enterovirus 71 Strains (EV71:TLLm and EV71:TLLmv). PLoS ONE 9(3): e92719 doi:10.1371/journal.pone.0092719 24671184 10.1371/journal.pone.0092719 PMC3966832

