# Supplementary figures and images for: Phenotypic and Genotypic Characteristics of Novel Mouse Cell Line (NIH/3T3)-Adapted Human Enterovirus 71 Strains (EV71:TLLm and EV71:TLLmv)
Source: PLoS One. 2014 Mar 26;9(3):e92719. doi: 10.1371/journal.pone.0092719 (PMC3966832; doi:10.1371/journal.pone.0092719)

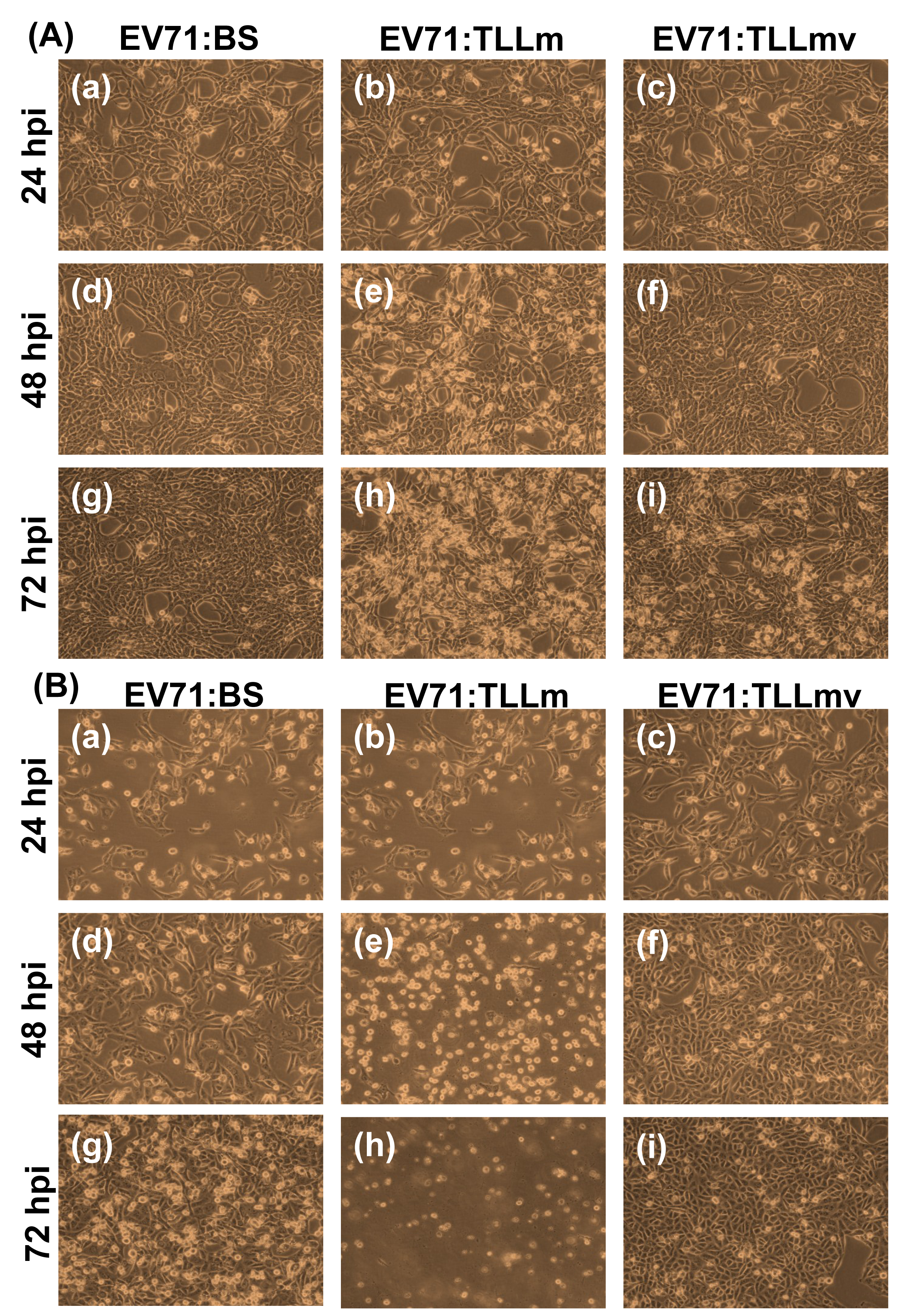

Supplement: Figure S1 — Virus fitness assessment of EV71:BS, EV71:TLLm, and EV71:TLLmv in NIH/3T3 and Vero cells at 30°C. Overnight seeded (A) NIH/3T3 and (B) Vero cells infected with EV71:BS (a, d, g), EV71:TLLm (b, e, h), or EV71:TLLmv (c, f, i) were incubated at 30°C and observed under the light microscope with phase-contrast at 24 hpi (a–c), 48 hpi (d–f), and 72 hpi (g–i). Images taken are representative of two independent experiments. (TIF) [file pone.0092719.s001.tif]

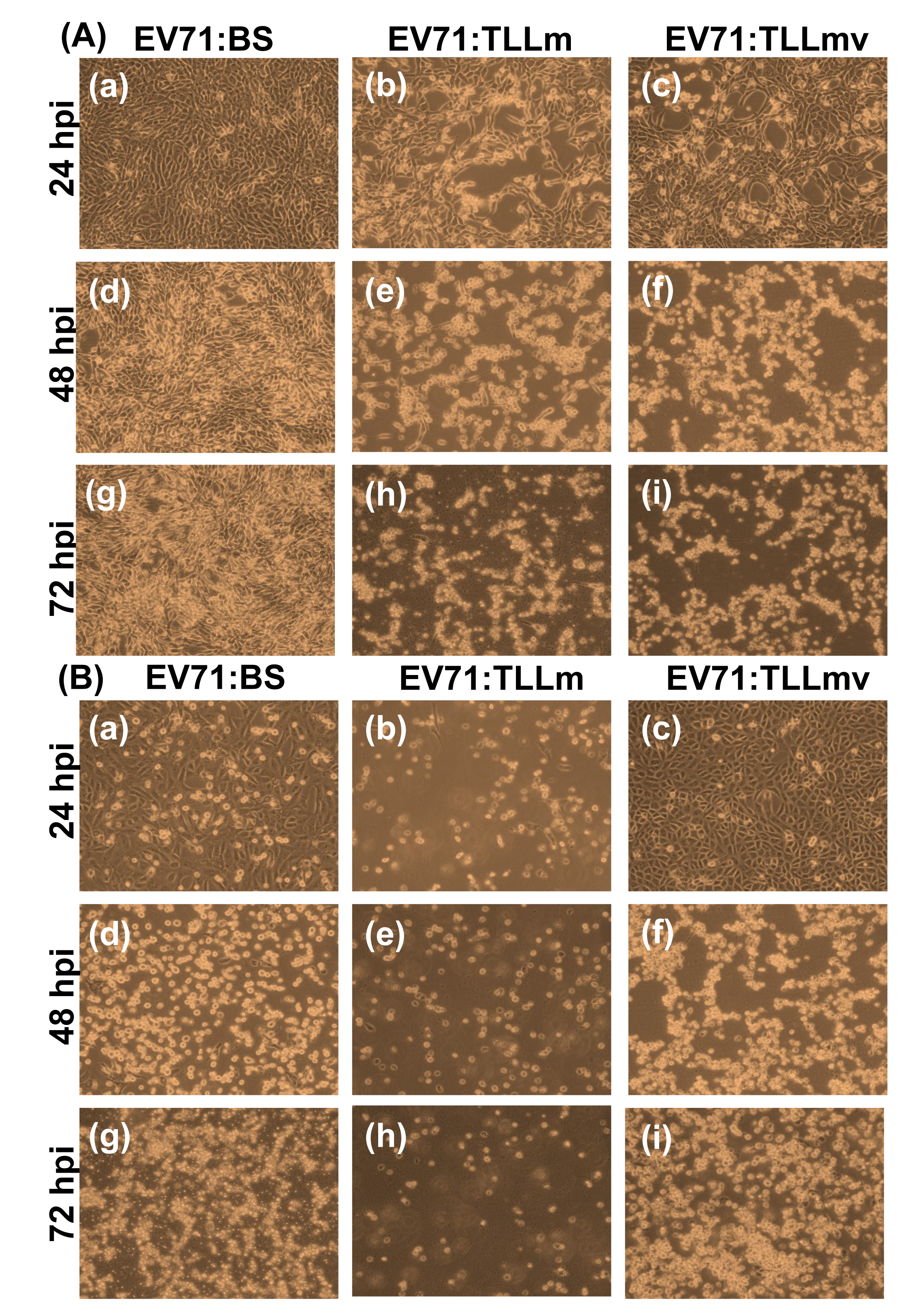

Supplement: Figure S2 — Virus fitness assessment of EV71:BS, EV71:TLLm, and EV71:TLLmv in NIH/3T3 and Vero cells at 37°C. Overnight seeded (A) NIH/3T3 and (B) Vero cells infected with EV71:BS (a, d, g), EV71:TLLm (b, e, h), or EV71:TLLmv (c, f, i) were incubated at 37°C and observed under the light microscope with phase-contrast at 24 hpi (a–c), 48 hpi (d–f), and 72 hpi (g–i). Images taken are representative of two independent experiments. (TIF) [file pone.0092719.s002.tif]

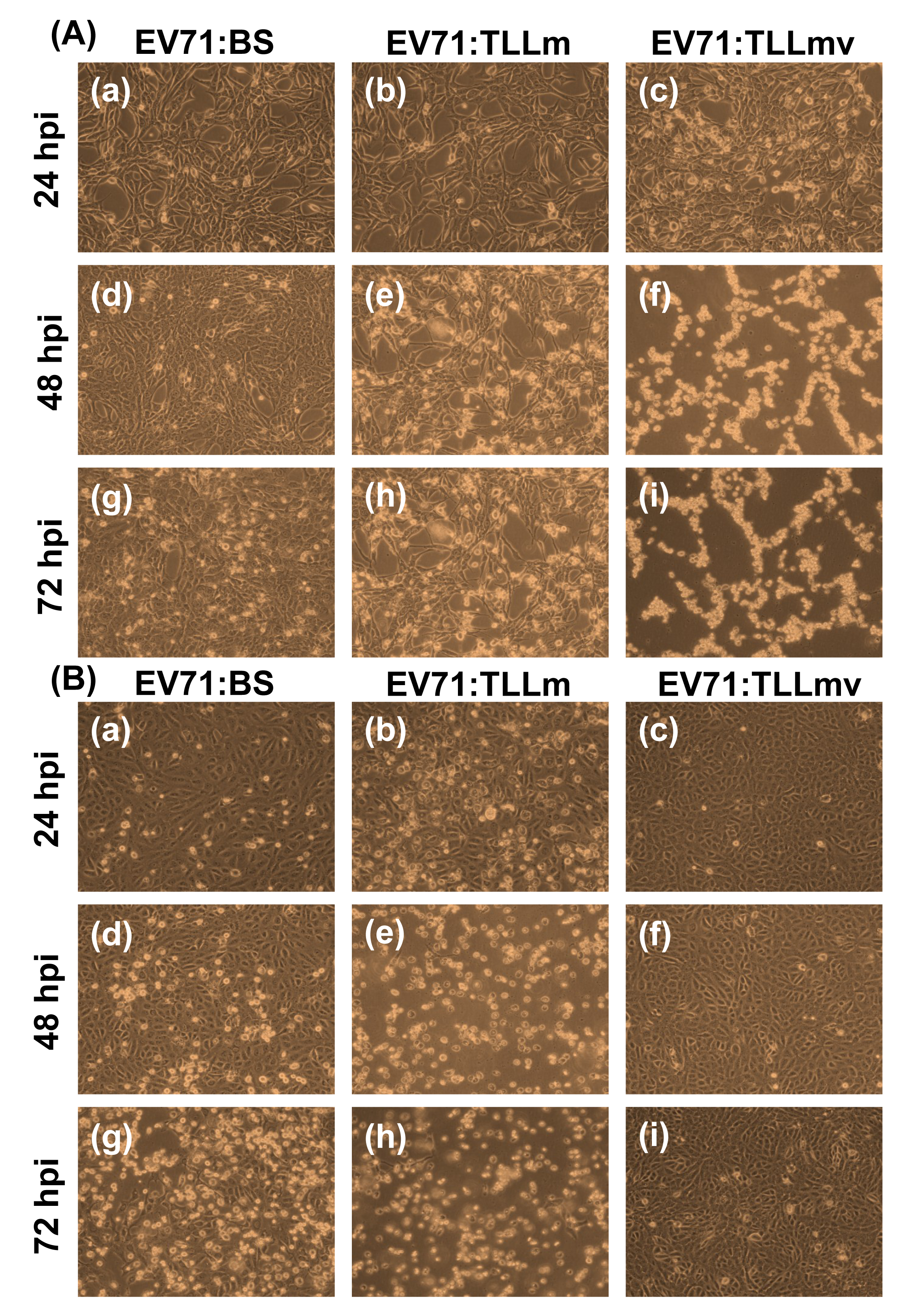

Supplement: Figure S3 — Virus fitness assessment of EV71:BS, EV71:TLLm, and EV71:TLLmv in NIH/3T3 and Vero cells at 39°C. Overnight seeded (A) NIH/3T3 and (B) Vero cells infected with EV71:BS (a, d, g), EV71:TLLm (b, e, h), or EV71:TLLmv (c, f, i) were incubated at 39°C and observed under the light microscope with phase-contrast at 24 hpi (a–c), 48 hpi (d–f), and 72 hpi (g–i). Images taken are representative of two independent experiments. (TIF) [file pone.0092719.s003.tif]

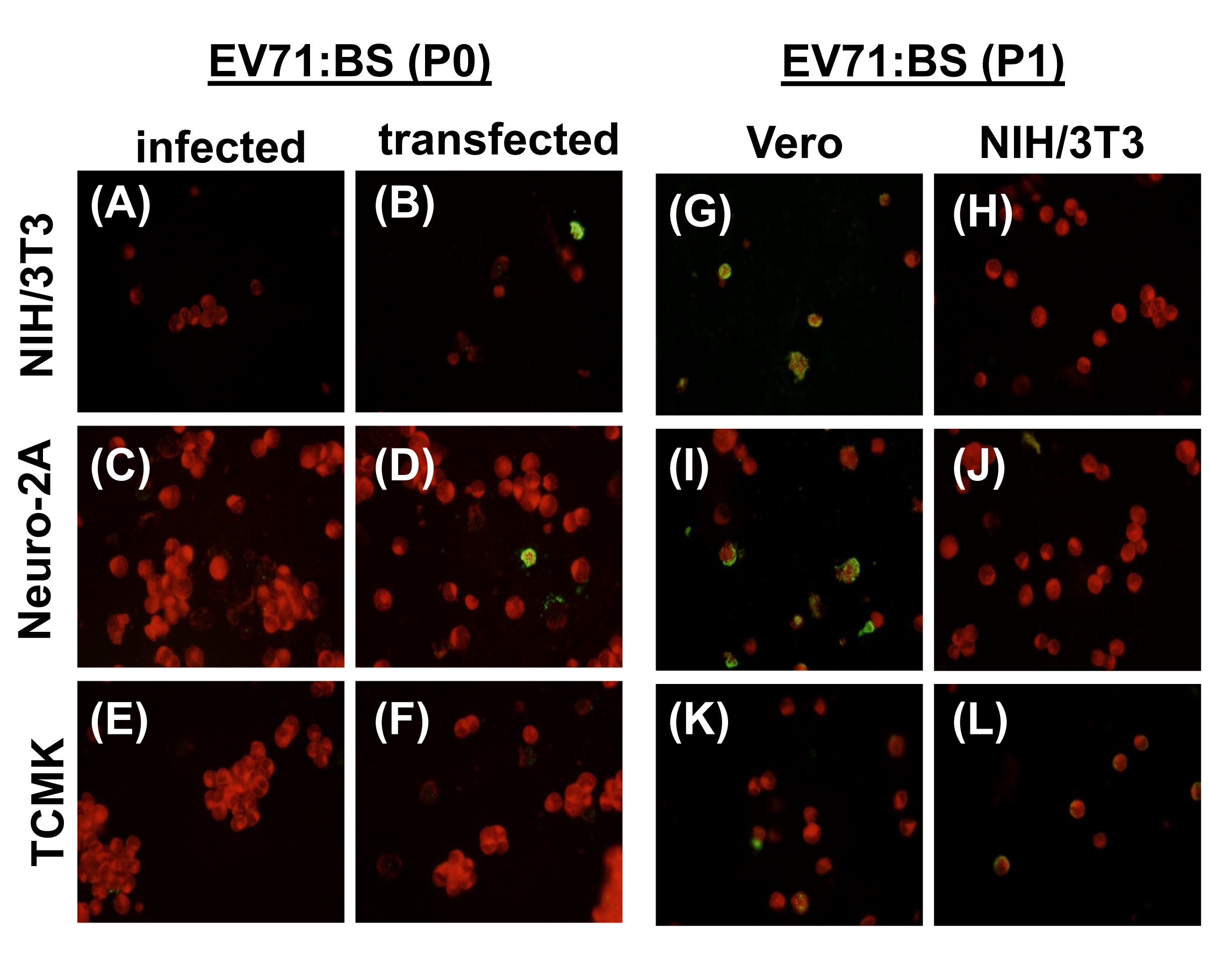

Supplement: Figure S4 — Transfection of murine cell lines NIH/3T3, Neuro-2A, and TCMK with EV71:BS viral RNA for evidence of virus replication. Overnight seeded NIH/3T3, Neuro-2A, and TCMK cells were either infected with 1000 CCID50 of EV71:BS virus (A, C, E) or transfected with equivalent amounts of viral RNA (B, D, F). and harvested at 48 hpi for viral antigen detection. Virus in the supernatants were harvested at 7 dpi and passaged onto fresh Vero (G, I, K) and NIH/3T3 cells (H, J, L). Cells were harvested and stained for viral antigens at 48 hpi. (TIF) [file pone.0092719.s004.tif]

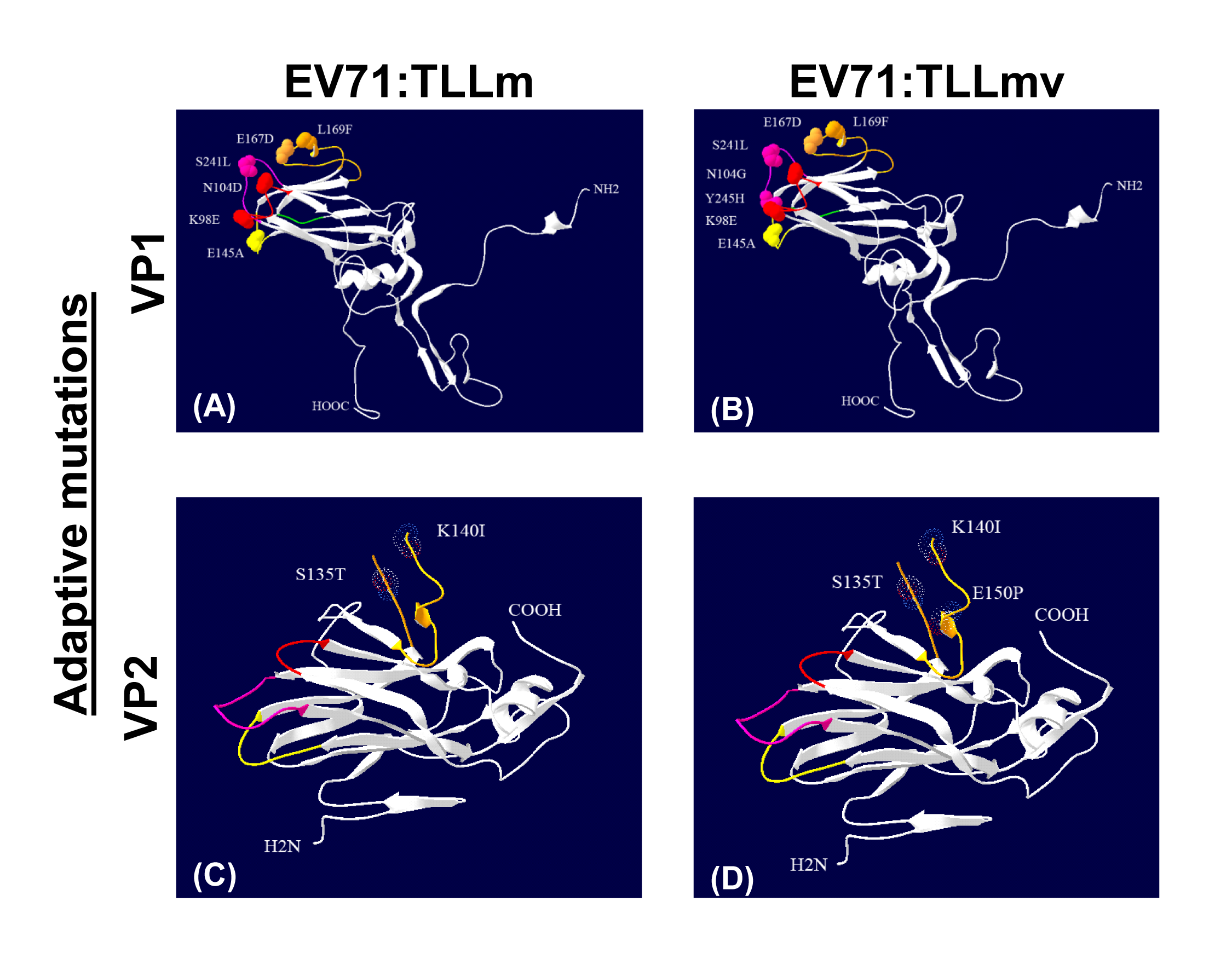

Supplement: Figure S5 — Localization in VP1 and VP2 of adaptive mutations in the genomes of EV71:TLLm and EV71:TLLmv. Adaptive mutations observed in the VP1 (A, B) and VP2 (C, D) regions of EV71:TLLm (A, C) and EV71:TLLmv (B, D) were modelled using DeepView/SwissPDBviewer v3.7 and the 3D structure of EV71 capsid P1 region (PDB ID 4AED). The mutations were observed to be mostly localized to the surface-exposed loops of the protein. The B–C loop is shown in red; D–E loop in yellow; E–F loop in orange; and G–H loop in pink. (TIF) [file pone.0092719.s005.tif]
